# Valorization of various nut residues grown in Turkiye: Antioxidant, anticholinesterase, antidiabetic, and cytotoxic activities^1^

**DOI:** 10.1002/fsn3.4103

**Published:** 2024-05-27

**Authors:** Gozde Kutlu

**Affiliations:** ^1^ Department of Gastronomy and Culinary Arts, Faculty of Fine Arts, Design and Architecture Ankara Medipol University Ankara Turkiye

**Keywords:** antioxidant activity, cytotoxic activity, enzyme inhibitory activity, nuts, shell and skin residues

## Abstract

The utilization of plant‐based residues has been extensively employed for the control of diverse illnesses, owing to their safety and minimal adverse effects. In the current study, it was aimed for the characterization of the bioactive, enzyme inhibitory, and cytotoxic activities of fresh pistachio shell skin (FPSS), green walnut husk and walnut membrane (GWH), almond outer shell and inner brown skin (ASIS), as well as peanut husk and inner skin (PHIS) to be used as industrial food processing by‐products. The results showed that the samples exhibited different extraction yields, with GWH having the highest percentage at 15.18%, followed by FPSS at 12.81%, ASIS at 10.29%, and PHIS at 7.80%. FPSS had the highest total phenolic content (16.28 mg gallic acid equivalents (GAE)/g) as well as the best antioxidant capabilities for DPPH (8.96 mg Trolox equivalent (TE)/g), FRAP (11.46 mg TE/g), and ABTS (22.38 mg TE/g) assays. FPSS was followed by PHIS, ASIS, and GWH, respectively. Moreover, the extracts exhibited relatively low activity against acetylcholinesterase, α‐glucosidase, and α‐amylase compared to standard acarbose or galantamine. Furthermore, the extracts may have the potential to induce cytotoxic effects, varying from moderate to mild, on both cancerous (IC_50_ = 454.55–617.28 μg/mL) and healthy cells (IC_50_ = 438.60–490.20 μg/mL). The results of this research showed that shell residues of nut hold promise for a variety of industrial applications spanning the food, pharmaceutical, and cosmetic sectors.

## Graphical abstract

Nut residual types significantly affected the bioactive properties. Extracts obtained from nut residues demonstrated cytotoxic impacts on MIA‐PaCa‐2 and CaCo‐2 cancer cells. Hazelnut residues exhibited inhibitory effects on enzymes, but these were less potent than the standards (galantamine or acarbose).

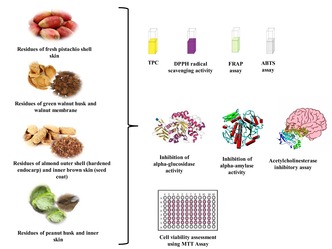

## 1 INTRODUCTION

Recently, the generation of by‐products has emerged as a major issue, leading to an increased focus on waste utilization strategies that are in line with circular economy principles (Kumar et al., 2017). Fruit husks have gained significant recognition as crucial biological assets for subsequent utilization in sectors such as the food industry, pharmaceutical manufacturing, and the production of bioenergy. Nevertheless, numerous fruit husk varieties remain underutilized within the realm of the food industry (Gharibi et al., 2023).

Pistachio (*Pistacia vera* L.), commonly known as the “green gold” and a member of the Anacardiaceae family, represents a traditional nut with substantial export, economic, and nutritional importance (Taghipour et al., 2023). The largest global pistachio production takes place in the United States of America, Turkiye, China, and Iran. Pistachio cultivation and the associated processes of removing the hulls involve generating a significant amount of organic waste. This waste includes the green hulls, the woody parts that hold clusters, shells, and leaves (Toghiani et al., 2023). The polyphenolic compounds present in pistachio hulls play significant roles in biofunctions like antioxidant and antimicrobial effects (Gharibi et al., 2023).

The walnut tree (*Juglans regia* L.), a renowned member of the Juglandaceae family, is globally cultivated. China accounts for approximately half of the world's walnut production, followed by the United States, Iran, and Turkiye. Walnut leaves and green husks, significant agricultural by‐products, draw attention due to their chemical composition encompassing protein, fat, carbohydrates, minerals (e.g., Zn, Mg, Fe, K, Ca, and Mn) and vitamin E, along with their bioactive components such as phenolics, triterpenic acids, terpenes, organic acids, flavonoids, and terpenoids. These materials, existing abundantly and inexpensively as waste, have the potential for the creation of high‐value products and functional foods. With attributes such as antiaging, antimicrobial, antioxidant, antihistaminic, anti‐inflammatory, antidiabetic, antidiarrheic, antiasthmatic, antiulcer, anticancer properties, walnut leaves and green husks could offer human health benefits (Fernández‐Agulló et al., 2013; Jahanban‐Esfahlan et al., 2019; Kafkas et al., 2020; Salik & Çakmakçi, 2023).

The almond tree (*Prunus amygdalus* L.), belonging to the Rosaceae family, is cultivated extensively in Turkiye, which holds the position of the world's fourth‐largest almond producer after the United States, Spain, and Iran (Gulsoy et al., 2022). Historically, this residual material has been used as animal feed and fuel or alternatively incinerated or disposed of in landfills, thus creating environmental problems (Valdés et al., 2022). The almond skin (33% of the total almond weight), called the endocarp, consists of densely packed lignocellulosic sclereid cells. It is mainly composed of cellulose, hemicellulose, and lignin (Ledbetter, 2008). The almond skin consists of components like total dietary fiber, soluble dietary fiber, lipids, and proteins. Despite constituting only about 4% of the total almond weight and having limited economic value, recent studies highlight that it contains approximately 60%–80% of the total phenolic compounds found in the nut. These phenolic compounds within the almond skin contribute significantly to its impressive radical scavenging activity, potentially contributing to the positive health effects associated with almond consumption (Prgomet et al., 2017).

Peanuts (*Arachis hypogaea* L.) are a globally important oilseed crop that is widely grown and valued. The processing of peanut oilseeds yields popular products but also important by‐products. These include peanut flour, shells, and hulls, which are primarily used as animal feed. Peanut shells, valuable by‐products from peanut processing, are particularly noteworthy, with annual global production exceeding 1 million tons. These shells have high concentrations of proanthocyanidins and their isomers (Sorita et al., 2023). Roughly 20% of the total weight of a whole peanut is attributed to its shells, which unfortunately do not have value‐added uses. The majority of these shells are disposed of, with only a portion being utilized in applications such as incorporating them into medium‐density fiberboards, using them as binding agents or plastic fillers, as solid fuel, or as fodder for livestock.

Considerable amounts of phytochemicals and bioactive substances have been discovered in various vegetable by‐products, including seeds, peels, leaves, and stems. These bioactive components form a valuable reservoir of molecules with significant potential for utilization as dietary supplements in food production, cosmetics, and pharmaceuticals. They offer numerous benefits, including accessibility, recyclability, affordability, eco‐friendly characteristics, absence of toxicity, and biodegradability. Recent literature contains numerous studies concerning the utilization of natural antioxidants (Valdés et al., 2022). In this context, the inclusion of nuts in a regular diet has been associated with potentially reduced susceptibility to certain diseases such as cancer and diabetes (Pinelo et al., 2004). Moreover, the aforementioned residual components of nuts could serve as an appealing and easily accessible natural antioxidants. They have the potential to be utilized as ingredients in the preparation of health‐enhancing foods or pharmaceutical products. Numerous studies in the literature explore the extraction, characterization, and effects of antioxidant compounds sourced from diverse origins. For instance, research has examined antioxidant compounds derived from almonds (Sarwar et al., 2012; Valdés et al., 2022), peanut shells (Adhikari et al., 2019), pistachio leaves and hulls (Elakremi et al., 2020; Gharibi et al., 2023), and walnut green husks (Fernández‐Agulló et al., 2013; Oliveira et al., 2008). Additionally, investigations have been conducted on enzyme inhibitory activities associated with peanut shell skin (Kilic et al., 2016), cytotoxic effects of peanut hulls on cancer cell lines including HeLa, MCF‐7, OE‐33, and ACC‐201 (Karaoglu & Tarhan, 2022; Seifaddinipour et al., 2018), and the impact of green walnut husk extracts on the proliferation and migration of gastric cancer cells (Zhang et al., 2022). However, there is no study that simultaneously examined and compared the cytotoxic and enzyme inhibitory properties of different nut residues in the same study. In order to fill this gap in the literature, the current study aimed to (i) assess the extraction yield, phenolic content, and antioxidant properties, (ii) determine enzyme inhibitory activities, and (iii) evaluate cytotoxic effects on human colorectal adenocarcinoma Caco‐2, human pancreas adenocarcinoma MIA PaCa‐2, and the human embryonic kidney cell line HEK‐293 using the ethanolic extract residues from fresh pistachio shell skin, green walnut husk, and walnut membrane, almond outer shell and inner brown skin, as well as peanut husk and inner skin.

## 2 MATERIALS AND METHODS

### 2.1 Chemicals and reagents

DPPH (2,2‐diphenyl‐1‐picrylhydrazyl), sodium carbonate (Na_2_CO_3_), and 2,2′‐azino‐bis‐(3‐ethylbenzothiazoline‐6‐sulfonic acid) (ABTS) were obtained from Sigma–Aldrich, Germany. All the used chemicals and reagents were analytical grade.

### 2.2 Collection of samples

In 2022, newly harvested green walnuts (*J. regia* L.) were obtained from Antalya, Turkiye during the month of August. Similarly, during the month of July, raw peanuts (*A. hypogaea* L.) and raw almonds (*P. amygdalus* L.) were sourced from the same region, Antalya, Turkiye. Additionally, fresh raw pistachios (*P. vera*) were procured from Gaziantep, Turkiye in 2022.

### 2.3 Preparation of extracts

The samples were manually deshelled, and the shells were stored at −80°C for 12 h before being processed using a laboratory freeze dryer (Martin Christ, Beta 1‐8 LSC plus, Osterode am Harz, Germany) (the absolute pressure: 13.33 Pa, condenser temperature: −48°C, the temperature of the heating plate: +10°C). The samples were then ground into a powdered form using a Tefal 8100.31 coffee grinder (France). For the extraction process, 500 g of the powdered samples was mixed with ethanol (Merck, 95%) in a ratio of 1:5 (w/v). The extraction was carried out using vacuum evaporation with a Buchi R‐220 system (Switzerland) for 6 h. After extraction, the mixture was centrifuged at 10,000 rpm and at room temperature for 10 min (Adhikari et al., 2019). The resulting supernatants obtained from fresh pistachio shell skin, green walnut husk and walnut membrane (1:1, w/w), almond outer shell and inner skin (1:1, w/w), and peanut husk and inner skin (1:1, w/w) were subsequently referred to as FBSS, GWH, ASIS, and PHIS extracts, respectively. Finally, extraction yields were calculated for each of them.

### 2.4 Determination of bioactive properties

#### 2.4.1 TPC

TPC of residual extracts was determined using the Folin–Ciocalteu colorimetric method, following the procedure detailed by Mollica et al. (2018). Initially, a test tube containing approximately 0.5 mL of 0.2 N Folin–Ciocalteu reagent, 0.5 mL of extracts, and 2 mL of Na_2_CO_3_ solution (7.5%, w/v) was prepared. The contents were thoroughly mixed and left to react in the dark at a temperature of 37°C for a duration of 30 min. Following this incubation period, absorbance measurements were taken at 760 nm using a spectrophotometer (Shimadzu UV‐1800, Nippon, Japan). The outcomes were then quantified as milligrams of gallic acid equivalents per gram of dry residual extract (mg GAE/g).

#### 2.4.2 Antioxidant activity assays

The nut extracts were subjected to assessment for their antioxidant capabilities using three distinct spectrophotometric techniques: 1,1‐diphenyl‐2‐picrylhydrazyl (DPPH) radical scavenging activity, ferric reducing antioxidant power (FRAP), and ABTS assays.

##### DPPH radical scavenging activity

At first, 1.5 mL of the ethanolic extract of nut residues was thoroughly mixed with an equal volume of DPPH solution. This mixture was vigorously vortexed for 1 min and then allowed to rest in a dark environment at room temperature for 30 min. Subsequently, measurements of absorbance were conducted at 517 nm employing a ultraviolet–visible spectrometer (Shimadzu UV‐1800, Kyoto, Japan) (Atlar et al., 2024; Mahomoodally et al., 2019; Subaşı‐Zarbaliyev et al., 2023).

##### FRAP assay

The assessment of antioxidant activity through the FRAP assay was conducted following the protocol outlined by Yasar et al. (2022). For each analysis, the FRAP reagent was freshly prepared by combining 300‐mM acetate buffer (pH: 3.6), 20‐mM ferric chloride solution, and a TPTZ solution (containing 10‐mM TPTZ in 40‐mM HCl) in proportions of 10:1:1. In the experimental procedure, 500 μL of FRAP solution and 20 μL of the nut residual extract were vigorously mixed and then allowed to incubate in the absence of light at room temperature for 8 min. Subsequently, absorbance measurements were taken at 593 nm. Ultimately, the obtained results were reported as milligrams of Trolox equivalent (TE) per gram on a dry weight basis (mg TE/g).

##### ABTS assay

The total antioxidant activity of residual extracts was assessed using the ABTS method, as outlined by Erol et al. (2023). A stock solution of ABTS^·+^ was generated by blending a 7‐mM ABTS solution with a 2.45‐mM potassium persulfate (K_2_S_2_O_8_) solution. This mixture was incubated in the dark at room temperature for 12–16 h. The resulting stock solution was then diluted in a 1:10 ratio (v/v) with 96% ethanol. For analysis, the sample extract (x mL) was combined with ethanol (4‐x mL) and subsequently mixed with the ABTS^·+^ solution. The reaction was allowed to proceed for 6 min, after which absorbance readings were taken at 734 nm using a spectrophotometer (Shimadzu UV‐1800, Nippon, Japan). The quantification of total antioxidant activity was expressed as milligrams of TE per gram of dried residual extract (mg TE/g) (Erol et al., 2023).

### 2.5 Determination of enzyme inhibitory activity

#### 2.5.1 Determination of in vitro antidiabetic activity

The investigation of potential antidiabetic effects of extracts was conducted using in vitro methodologies, specifically targeting the activity of α‐amylase and α‐d‐glucosidase enzymes. This approach followed the methodology previously applied by Erol et al. (2023). A comparison was made against the curve of acarbose, a synthetic drug widely utilized for diabetes management due to its α‐glucosidase inhibitory properties.

##### Inhibition of α‐glucosidase activity

In this method, galantamine was used as the positive control, while buffer served as the blank. For this procedure, a 25‐μL aliquot of the sample, previously diluted in buffer, was combined with 25 μL of α‐glucosidase (0.5 U/mL). This mixture was preincubated at 25°C for 10 min before introducing 25 μL of 0.5 mM para‐nitrophenyl‐α‐d‐glucopyranoside (P‐NPG) to each well as the substrate. Following a 10‐min incubation at 37°C, the reaction was halted by adding 100 μL of 0.2 M Na_2_CO_3_ to the mixture. The absorbance of resulting solution was then measured spectrophotometrically at 405 nm.

##### Inhibition of α‐amylase activity

For this investigation, a blend consisting of 50 μL of samples diluted with buffer was prepared. Following that, the enzyme α‐amylase (25 μL, 0.5 U/mL) was allowed to preincubate at 25°C for 10 min, after which 50 μL of a 0.5% fresh starch solution (w/v) was added to each well. Subsequently, the mixture was subjected to an additional 10‐min incubation at 25°C. Following this, a 100‐μL volume of 1% DNSA (3,5‐dinitrosalicylic acid) reagent was incorporated into the mixture, and the resulting solution was kept at a temperature of approximately 85–90°C for 10 min using a hot water bath. Lastly, the cooled samples were assessed for absorbance at 405 nm using a spectrophotometer.

#### 2.5.2 Determination of anti‐Alzheimer's disease activity

##### Acetylcholinesterase (AChE) inhibitory assay

In this examination, AChE obtained from electric eel was employed as the source of the enzyme. To accomplish this, a 20‐μL solution of the sample, which was diluted with buffer from a 50 mg/mL DMSO stock solution, was combined with 140 μL of phosphate buffer (0.1 mM, pH 6.8). To this mixture, 20 μL of AChE (5 × 10^−3^ M) enzyme solution was added, and the mixture was left to incubate for approximately 10 min. The reaction was then initiated by introducing 10 μL of 3 mM DTNB (5,5′‐dithiobis‐(2‐nitrobenzoic acid)) and 10 μL of 0.71 mM acetylcholine iodide (AChI) to the solution. The absorbance of the reaction mixture at 405 nm was measured using a microplate reader (Epoch, USA) (Erol et al., 2023).

### 2.6 In vitro cytotoxicity activity

#### 2.6.1 Cell culture

The cytotoxic effects of nut residues were examined using three distinct cancer cell lines along with a healthy cell line. The cancer cell lines included human colorectal adenocarcinoma CaCo‐2 (ATCC, #HTB‐37) and human pancreas adenocarcinoma MIA PaCa‐2 (ATCC, CRL‐1420). Additionally, the human embryonic kidney cell line HEK‐293 (ATCC, CRL‐1573) was used as the healthy cell. All these cell lines were acquired from the American Collection of Cell Cultures and cryopreserved in liquid nitrogen prior to culturing.

The cells were cultivated in Dulbecco's modified Eagle's medium (DMEM) supplemented with 10% fetal bovine serum (FBS), 1% l‐glutamine, and 1% antibiotics (penicillin/streptomycin). The incubation was carried out at 37°C within a humidified atmosphere composed of 5% CO_2_ and 95% air. The cells were grown in plastic flasks of either 25 or 75 cm^2^ surface area.

#### 2.6.2 Cell viability assessment using MTT assay

For this aim, in a nutshell waste, 15 × 10^3^ cells per well were placed into 96‐well plates and cultured for 24 h. Following the removal of the culture medium, varying concentrations of cell‐free filtrate and cell‐free lyophilized filtrate were introduced. As a negative control, noninoculated DMEM F‐12 medium was utilized. For the CaCo‐2 cells, a further incubation span of 8 and 24 h was conducted. Subsequently, MTT solution (100 μL/mL) was incorporated into each well. After a 2‐h incubation period at 37°C, 100 μL of dimethyl sulfoxide (DMSO) was added to dissolve the resultant blue crystals, and absorbance measurements were taken. A microplate reader (Bio‐Tek, ELX808IU, USA) was utilized to determine the optical density at 570 nm.

### 2.7 Statistical evaluation

Data was presented as mean ± standard deviation. JMP 6.0 software (SAS Institute, Inc., Cary, USA) was used to make comparisons between outcomes and mean experimental values using one‐way ANOVA (analysis of variance). In addition, Student's *t*‐test was used to determine differences between the results at a significance threshold of 0.05 (*p* ≤ .05).

## 3 RESULTS AND DISCUSSION

### 3.1 Extraction yield

As seen in Table 1, the extraction yield of the nut residues ranged between 7.80% and 15.18% and showed statistically significant differences (*p* < 0.05). The extraction yields of the samples were ranked from most to least as follows: GWH (15.18%) > FPSS (12.81%) > ASIS (10.29%) > PHIS (7.80%). Previously, Oliveira et al. (2008) reported that the extraction yield values of GWH from Parisienne, Mayette, Marbot, Franquette, and Mellanaise varieties were between 31.63% and 33.90%. Subsequently, it was reported that the extraction yield of GWH varied from 3.90% (ethanolic extract) to 44.11% (aqueous extract) depending on the extraction solvent (Fernández‐Agulló et al., 2013). Additionally, Kilic et al. (2016) reported that the determination of methanol extract yields from both immature and mature shell skins of *P. vera* resulted in percentages of 12.23% (w/w) and 36.11% (w/w), respectively. Furthermore, Queirós et al. (2020) reported that the ethanol–water extracts of walnut, almond, and pine nutshells were 7.3%, 3.4%, and 3.9%, respectively. The yield of the extract was influenced by various factors such as climate, plant species, extraction temperature, extraction time, solid: solvent ratio, soil characteristics, harvest phase, solvent type, pH, geographical location, and extraction methodology (Fernández‐Agulló et al., 2013; Karaoglu & Tarhan, 2022; Yasar et al., 2022).

**TABLE 1:** Extraction yield and bioactive properties of various nut residues.

Type of wastes	Abbreviation of waste types	Extraction yield (%)	TPC (mg GAE/g)	Antioxidant activities		
				DPPH (mg TE/g)	FRAP (mg TE/g)	ABTS (mg TE/g)
Fresh pistachio shell skin	FPSS	12.81 ± 0.01^b^	16.28 ± 0.13^a^	8.96 ± 0.05^a^	11.46 ± 0.08^a^	22.38 ± 0.10^a^
Green walnut husk	GWH	15.18 ± 0.00^a^	0.81 ± 0.02^d^	0.40 ± 0.01^d^	0.61 ± 0.02^d^	0.90 ± 0.02^d^
Almond shell and inner skin	ASIS	10.29 ± 0.01^c^	3.19 ± 0.07^c^	1.00 ± 0.05^c^	1.50 ± 0.02^c^	4.47 ± 0.02^c^
Peanut husk and inner skin	PHIS	7.80 ± 0.01^d^	4.27 ± 0.10^b^	2.41 ± 0.02^b^	2.90 ± 0.04^b^	6.87 ± 0.04^b^

Abbreviations: ABTS, ABTS (2,2′‐azino‐bis (3‐ethylbenzothiazoline‐6‐sulfonic acid)) antioxidant activity; DPPH, DPPH (2,2‐diphenyl‐1‐picrylhydrazyl) antioxidant activity; FRAP, ferric reducing antioxidant power antioxidant activity; GAE, gallic acid equivalent; TE, trolox equivalent; TPC, total phenolic content. If different letters are used to denote values in the same column, this indicates significant differences (*p* < 0.05).

### 3.2 Bioactive properties

The data regarding the bioactivity measurements of different nut residues were listed in Table 1. The results displayed a significant range in TPC, ranging from 16.28 to 0.81 mg GAE/g, with notable statistical significance (*p* < 0.05). Similar findings were reported for pure and aqueous methanolic and ethanolic extracts from thick and thin shell almond (1.36–7.21 mg GAE/g) (Sarwar et al., 2012), ethanol extract (2.31–7.21 mg GAE/g), and methanol extract (1.06–4.12 mg) from almond hulls (Pinelo et al., 2004). However, lower TPC results were recorded for methanolic extract of peanut shell (0.4281–0.7398 mg GAE/g) (Adhikari et al., 2019). In contrast, higher TPC results were recorded for ethanol–water extracts of shells from walnuts (317.9 mg GAE/g extract) (Queirós et al., 2020) and almonds (188.6 mg GAE/g extract) (Queirós et al., 2020) as well as ethanolic extract of pistachio shells (189 mg GAE/g extract) (Cardullo et al., 2021). The wide range of results in the literature is due to the fact that TPC values are affected by the difference in extraction methods, the type and amount of sample used, and the type of solution used in the extraction.

Due to the intricate nature of oxidation and antioxidation, no single technique can precisely and fully assess the antioxidant effects of diverse natural substances and plant chemicals. Numerous theories exist regarding how antioxidants function, including stopping chain reactions caused by free radicals, binding to catalytic ions, providing hydrogen atoms, and removing peroxides (Yasar et al., 2022). Consequently, employing several approaches to gauge antioxidant properties of various nut residues became crucial. The scavenging activity of various nut residues against DPPH radicals exhibited considerable diversity, with values ranging from 0.40 to 8.96 mg TE/g, and these differences were statistically significant (*p* < 0.05). The considerable ability of peanut shells to scavenge DPPH radicals could be attributed to the existence of diverse antioxidant compounds (Adhikari et al., 2019). The study identified a positive correlation between TPC and DPPH radical scavenging activities in the nut residues.

The FRAP activities of various nut residues are also given in Table 1, demonstrating variation based on the source of nut residue. Generally, FRAP activity tended to align with TPC, which was in line with the findings of Fernández‐Agulló et al. (2013). For instance, the sample with the lowest TPC (0.81 mg GAE/g), namely, GWH, displayed the lowest FRAP activity (0.61 mg TE/g). Conversely, the sample with the highest TPC (16.28), FPSS, exhibited the highest FRAP activity (11.46 mg TE/g). The substantial phenolic content might contribute to the notable antioxidant capacities of FPSS, positioning them as a promising natural source of antioxidants. In a related study, hydrolyzable tannins, flavonoids, and phenolic acids, along with their derivatives were identified as the primary components in the fractions displaying the most potent antioxidant activity in pistachio hard shells (Cardullo et al., 2021). Tomaino et al. (2010) reported that cyanidin‐3‐*O*‐galactoside, catechin, gallic acid, eriodictyol‐7‐*O*‐glucoside, and epicatechin had a significant role in the antioxidant properties of pistachio skin.

In terms of ABTS radical scavenging activity, the recorded values ranged from 0.90 (GWH) to 22.38 mg TE/g (FPSS), underlining a broad spectrum of antioxidant potency among nut by‐products, as evident from Table 1. The elevated ABTS radical scavenging activity observed in FPSS could be attributed to the presence of phenolic compounds.

### 3.3 Enzyme inhibitory activity

AChE, a type of cholinesterase, is a primary enzyme responsible for hydrolyzing acetylcholine (ACh), reducing essential neurotransmission levels and contributing to neurodegenerative disorders like Alzheimer's disease, making its inhibition a potential remedy for Alzheimer's (Tamfu et al., 2022). The inhibitory activities of various nut residues on AChE, α‐glucosidase, and α‐amylase are presented in Table 2. Notably, the AChE inhibition at a concentration of 2 mg/mL was recorded as 53.25%, 65.77%, 50.14%, and 40.93% for FPSS, GWH, ASIS, and PHIS, respectively. However, the standard drug galantamine exhibited more pronounced inhibition of AChE (85.35%) compared to the nut residues.

**TABLE 2:** Enzyme inhibition activities of various nut residues.

Type of wastes	Anticholinesterase activity	Antidiabetic activity	
	AChE (%)	α‐Glucosidase (%)	α‐Amylase (%)
FPSS	53.25 ± 0.16^c^	77.51 ± 0.16^b^	59.81 ± 0.15^b^
GWH	65.77 ± 0.17^b^	74.53 ± 0.10^c^	63.73 ± 0.09^c^
ASIS	50.14 ± 0.11^d^	51.39 ± 0.04^d^	40.87 ± 0.06^d^
PHIS	40.93 ± 0.12^e^	42.28 ± 0.07^e^	34.72 ± 0.04^e^
Galantamine	85.35 ± 0.12^a^	—	—
Acarbose	—	90.68 ± 0.09^a^	88.16 ± 0.14^a^

Abbreviations: AchE, acetylcholinesterase; ASIS, almond shell and inner skin; FBSS, fresh pistachio shell skin; GWH, green walnut husk and walnut membrane; PHIS, peanut husk and inner skin. If different letters are used to denote values in the same column, this indicates significant differences (*p* < 0.05).

α‐Glucosidase breaks down complex carbohydrates in the small intestine and inhibiting it can help control postprandial glucose levels without causing weight gain or hypoglycemia risks, while α‐amylase also holds importance in diabetes therapy (Gulçin et al., 2018). Regarding α‐glucosidase inhibition, FPSS demonstrated an activity of 77.51%, followed by GWH at 74.53%, ASIS at 51.39%, and PHIS at 42.28%. Similarly, for α‐amylase inhibition, FPSS exhibited 59.81%, GWH showed 63.73%, ASIS displayed 40.87%, and PHIS presented 34.72% activity. Acarbose, a widely employed α‐glucosidase inhibitor in type 2 diabetes mellitus therapy (Erol et al., 2023), exhibited higher inhibitory activity against both α‐glucosidase (90.68%) and α‐amylase (88.16%) compared to the tested extracts. These findings suggest that the extracts displayed relatively lower activity against AChE, α‐glucosidase, and α‐amylase. Result variations might be attributed to the diverse mechanisms through which the enzymes interact with the tested extracts (Orhan et al., 2004).

### 3.4 Cytotoxic activity

**FIGURE 1:**
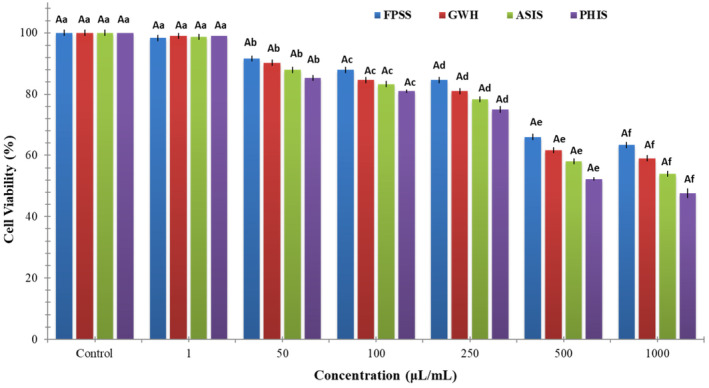
In vitro cytotoxic activity of various nutshells on the CaCo‐2 cell line. Different letters denote statistically significant differences (*p* < 0.05). An uppercase letter signifies statistical differences among various nut residues at the same concentration, while a lowercase letter indicates statistical differences among different concentrations of the same nut residues.

**FIGURE 2:**
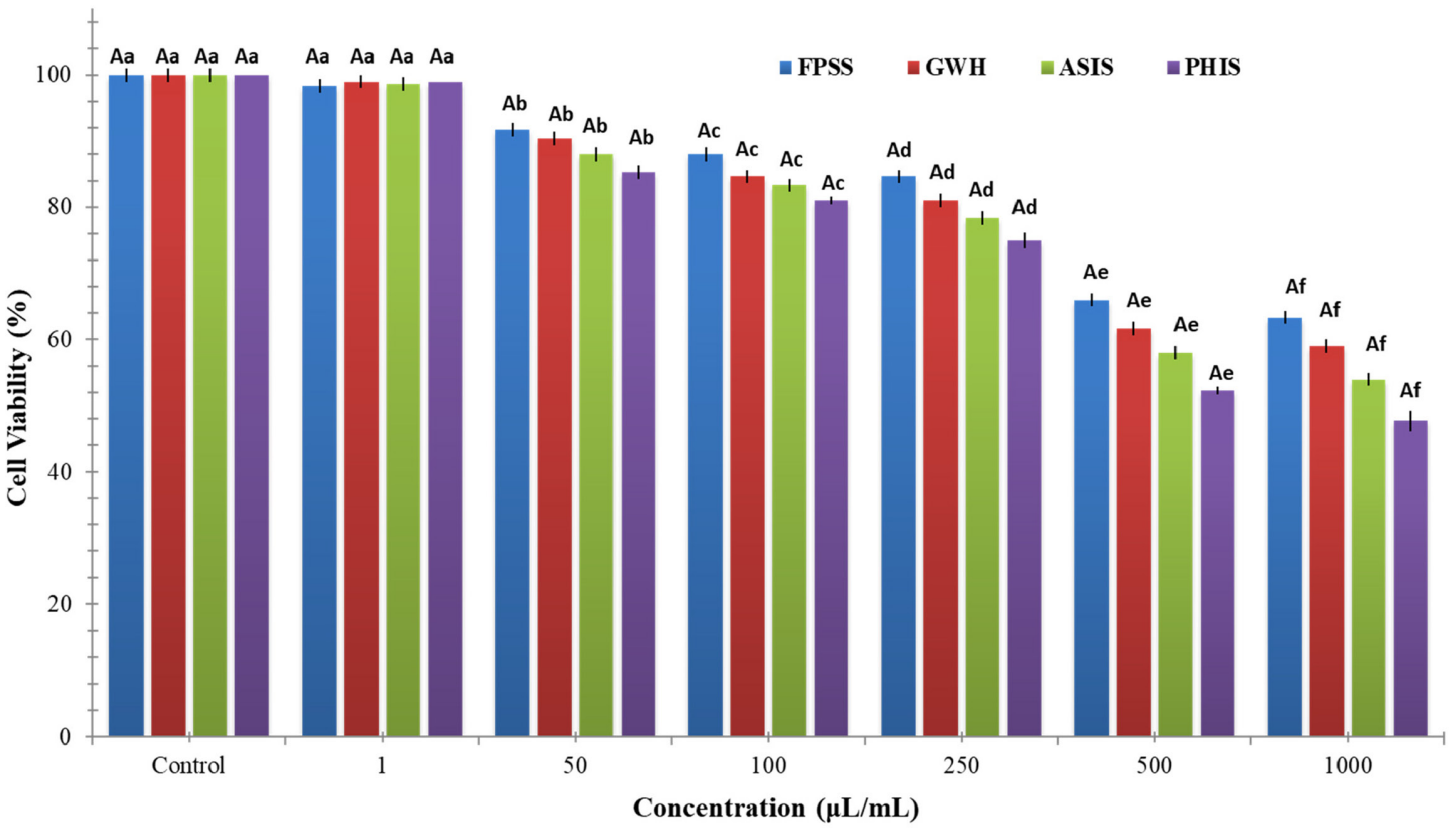
In vitro cytotoxic activity of various nutshells on the MIA‐PaCa‐2 cell line. Different letters denote statistically significant differences (*p* < 0.05). An uppercase letter signifies statistical differences among various nut residues at the same concentration, while a lowercase letter indicates statistical differences among different concentrations of the same nut residues.

**FIGURE 3:**
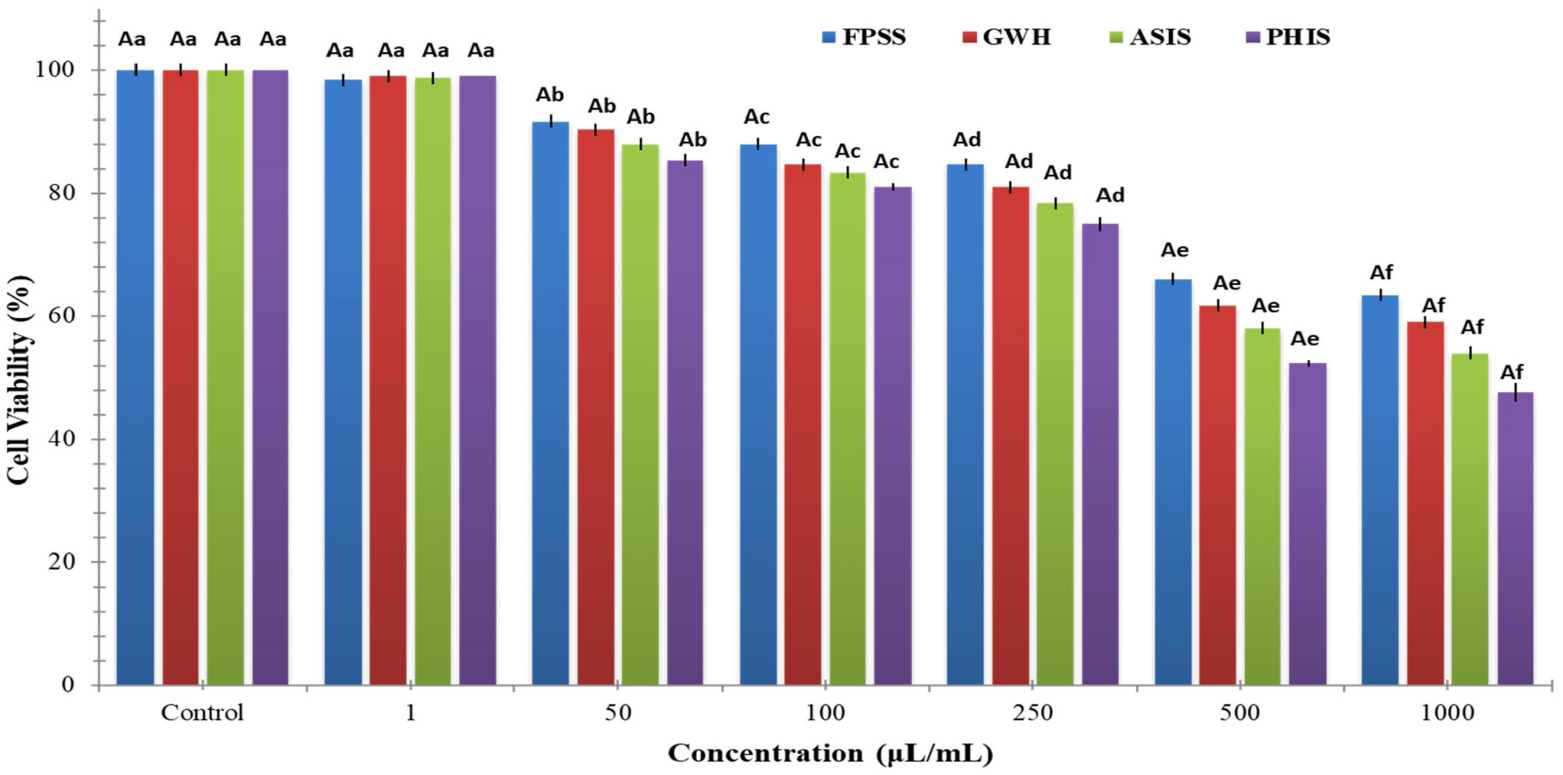
In vitro cytotoxic activity of various nutshells on the HEK‐293 cell line. Different letters denote statistically significant differences (*p* < 0.05). An uppercase letter signifies statistical differences among various nut residues at the same concentration, while a lowercase letter indicates statistical differences among different concentrations of the same nut residues.

The nut residues were assessed for their cytotoxic effects on colorectal adenocarcinoma cell line CaCo‐2, pancreatic adenocarcinoma cell line MIA PaCa‐2, and human embryonic kidney cell line HEK293. After 24 h, the viability of CaCo‐2 cells treated with nut residues at a concentration of 1000 μg/mL ranged between 64.33% and 73.33%. The findings indicated no statistically significant distinction among the samples in regard to cell viability. As illustrated in Figure 1, each of nut residues demonstrated a decrease in cell viability that was dependent on the concentration. Based on the MTT assay findings, the samples displayed an inhibitory impact on MIA‐PaCa‐2 cells (Figure 2). The cell viability percentage varied from 47.67% to 63.33% for the four nut residues at a concentration of 1000 μg/mL after 24 h of incubation. Numerous research studies have indicated that the utilization of natural antioxidants can lower the likelihood of diverse ailments, including cancers. These beneficial impacts have primarily been linked to the existence of phenolic compounds and flavonoids. These compounds possess the ability to neutralize cancer‐causing substances and hinder the activities of enzymes accountable for neutralizing carcinogens (Nile et al., 2018). Moreover, as depicted in Figure 3, the four samples of nut residues demonstrated a reduction in cell viability that was dependent on the concentration. All hydrolysates revealed minimal cytotoxic impact on the cell proliferation of the HEK‐293 cell line. The least cell viability (67.33%) was observed with PHIS at a concentration of 1000 μg/mL following 24 h of incubation, succeeded by 70.33% for ASIS, 74.33% for GWH, and 76.67% for FPSS.

The IC_50_ value, which stands for the half‐maximal inhibitory concentration, represents the concentration of a substance or extract required to inhibit 50% of the growth of a cell line. IC_50_ values for FPSS, GWH, ASIS, and PHIS against CaCo‐2 cell line at 24 h were determined as 454.55, 471.70, 490.20, and 515.46 μg/mL, respectively. Moreover, the IC_50_ values for MIA PACA‐2 cell line at 24 h were found to be 505.05 μg/mL for FPSS, 531.91 μg/mL for GWH, 568.18 μg/mL for ASIS, and 617.28 μg/mL for PHIS. Additionally, the IC_50_ values for FPSS, GWH, ASIS, and PHIS against the HEK‐293 cell line after 24 h were measured as 438.60, 450.45, 471.70, and 490.20 μg/mL, respectively. Typically, lower IC_50_ values indicated higher cytotoxic activity, whereas higher IC_50_ values suggested lower cytotoxic activity. In the plant screening program conducted by the National Cancer Institute of the United States, a raw extract was typically classified as having cytotoxic activity in vitro if its IC_50_ was less than 30–40 μg/mL (Oskoueian et al., 2011). Taking this criterion into account, it appeared that the extracts might possess moderate to low cytotoxic effects against both cancerous and healthy cells. Also, Bekir et al. (2013) indicated that the substantial levels of total phenolics present in polar extracts predominantly contributed to their cytotoxic effects.

Similarly, Zhang et al. (2022) investigated the effectiveness of GWH extracts against gastric cancer. They assessed the impact on SCG7901 cell proliferation using CCK‐8 and colony formation tests, observing a significant decrease in cell viability as GWH extract concentrations increased (10–200 μg/mL). Extracts at 100 μg/mL demonstrated a substantial reduction of approximately 50% in cell viability based on CCK‐8 assay. Moreover, Karaoglu and Tarhan (2022) studied on cytotoxic activities of pistachio (*P. vera* L.) hull samples from Turkiye against HeLa (IC_50_ = 25 ppm), MCF‐7 (IC_50_ = 25 ppm), OE‐33 (IC_50_ = 40 ppm), and ACC‐201 (IC_50_ = 28 ppm) cancer cell lines. Additionally, Seifaddinipour et al. (2018) focused on the cytotoxic effects of pistachio (*P. vera* L.) hulls and reported that IC_50_ values against MCF‐7 is 44.88 μg/mL.

## 4 CONCLUSIONS

Peanut, pistachio, and almond shells as well as green walnut shell and walnut membrane, typically discarded as waste, exhibited remarkable antioxidant abilities (measured by ABTS DPPH, and FRAP activity) accompanied by significant levels of total polyphenols. Among the various hazelnut residues tested, FPSS exhibited the highest TPC and had superior DPPH, FRAP, and ABTS antioxidant capacities. These findings emphasize that FPSS is most effective in scavenging free radicals and position it as a valuable potential source of potent antioxidants. Furthermore, the samples showed different extraction yields, with green walnut shell and walnut membrane having the highest percentage, followed by pistachio shell, almond shell, and peanut shell, respectively. All hazelnut residues demonstrated inhibitory activities against α‐amylase, α‐glucosidase, AChE, and BuChE; nevertheless, the observed effects were lower compared to the standards (galantamine/acarbose). The extracts could potentially exhibit cytotoxic effects ranging from moderate to mild on both cancer and noncancer cells. The findings from this study indicated that the residues of these nut shells have the potential for a variety of industrial uses covering the food, pharmaceutical, and cosmetic fields.

## AUTHOR CONTRIBUTIONS

**Gozde Kutlu:** Investigation (lead); methodology (lead); writing – original draft (lead); writing – review and editing (lead).

## CONFLICT OF INTEREST STATEMENT

The author declares no conflict of interest regarding the publication of this manuscript.

## ETHICS STATEMENT

This research does not have any ethical issues.

## Data Availability

Data are available on request from the author.
